# Strontium Lactate in Tænia

**Published:** 1892-08

**Authors:** 


STRONTIUM LACTATE IN TÆNIA.—Laborde ("*Journal de Med. de Paris*") has had excellent results in tænia with the usual dietary care from the following:

R Strontii Lact. (Paraf-Javal)...................dr. j
Aquæ ..........................................dr. viij
Glycerini..................... ................q. s.
M. S. Two teaspoonfuls every morning for five days.

This is practically the same strength as the standard solutions of Stron: Lact: (Paraf-Javal) used so largely in albuminuria.—*Medical Standard.*

